# *In silico* analyses of isoniazid and streptomycin resistance-associated mutations in *Mycobacterium tuberculosis*

**DOI:** 10.1016/j.csbj.2023.02.035

**Published:** 2023-02-21

**Authors:** Rushikesh Singh Dasoondi, Tom L. Blundell, Arun Prasad Pandurangan

**Affiliations:** Department of Biochemistry, University of Cambridge, United Kingdom

## Abstract

Multi-drug resistant tuberculosis is categorised by the World Health Organisation (WHO) as a public health crisis. *In silico* techniques were used to probe the structural basis of *Mycobacterium tuberculosis* resistance to isoniazid and streptomycin. Isoniazid resistance-associated mutations in InhA were predicted to reduce the binding affinity of NADH to InhA, without affecting INH-NAD (competitive-inhibitor) binding. Perturbation of the mutated residues was predicted (with the AlloSigMA server) to modulate the free energy of allosteric modulation of key binding site residues F41, F149, Y158 and W222. These results suggest that allosteric modulation of the protein structure may be key to the mechanism by which isoniazid resistance-associated mutations act. Mutations in the methyltransferase glucose-inhibited division gene B (GidB) are associated with streptomycin resistance. Molecular docking was carried out to predict the structure of the GidB bound to its substrate (s-adenosyl methionine). The effects of streptomycin resistance-associated mutations in GidB on protein stability and substrate binding were predicted (using SDM and mCSM-lig). All GidB mutants were predicted to disfavour SAM binding.

## Graphical Abstract

**Figure:**
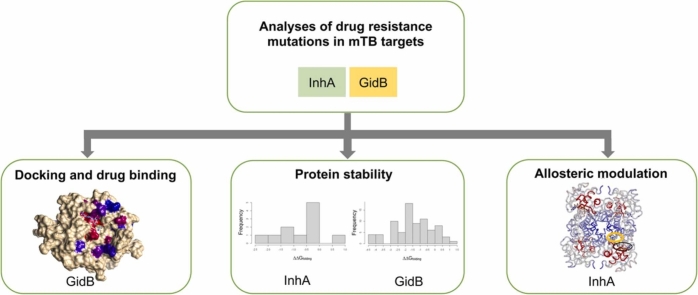
In silico analyses of isoniazid and streptomycin resistance-associated mutations in Mycobacterium tuberculosis drug targets InhA and GidB.

## 1 Introduction

In 2020, *Mycobacterium tuberculosis* (Mtb) was the world’s second most deadly infectious pathogen, preceded only by SARS-CoV-2 [1]. Consequently, the prevalence of drug-resistant (DR) Mtb strains presents a significant challenge. In 2018, the treatment success rate in patients infected with DR-TB was reported at 55%, compared to 85% for drug-susceptible tuberculosis (DR-Mtb) [2]. Among the most prevalent DR-Mtb strains are those resistant to isoniazid and streptomycin. A recent review of antibiotic resistant Mtb strains in Bangladesh [3] found that isoniazid and streptomycin resistant strains were most prevalent of all drug-resistant strains in previously treated patients (49.9% and 42.5%, respectively). This is disconcerting, since isoniazid forms the basis for first-line treatment of tuberculosis [4]. Moreover, streptomycin was recently reclassified as a second-line drug by the WHO, in part due to the high prevalence of resistant Mtb strains identified in newly diagnosed patients [5], [6].

**Fig. 1:**
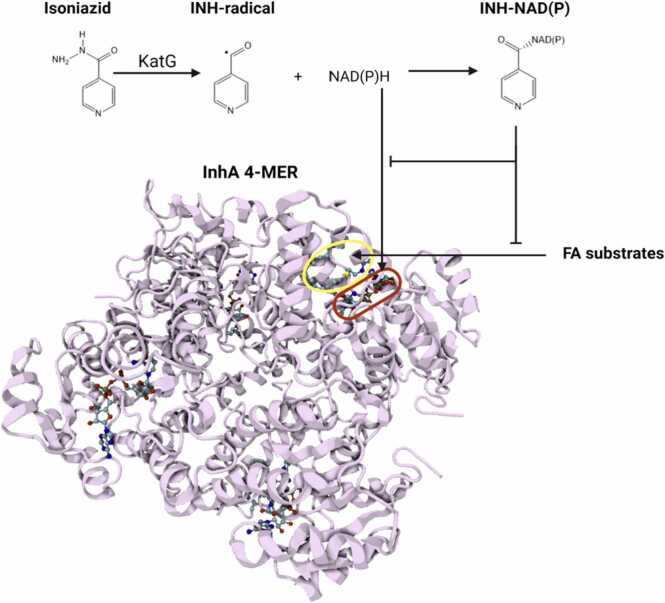
Isoniazid mechanism of action. Isoniazid is converted to the active radical form by the enzyme KatG. The active form reacts with NAD(P)H to form a covalent adduct, which acts as a competitive inhibitor of NADH cofactor (circled in red) and fatty acid/FA substrate (circled in yellow) binding.

**Fig. 2:**
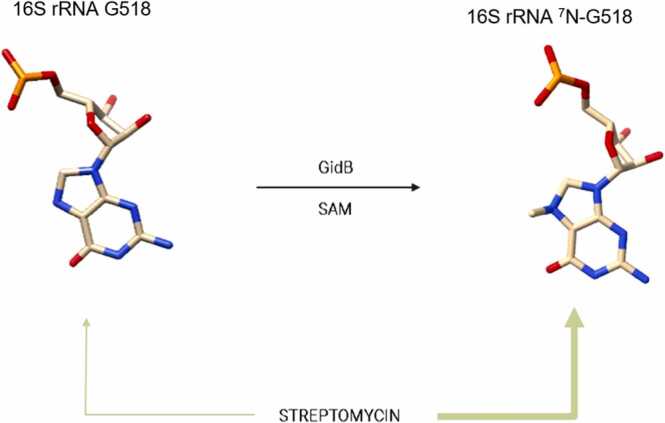
Methylation of 16 S rRNA ^7^N-G518 by GidB. GidB methylates ^7^N-G518 of the 16 S rRNA using substrate SAM as methyl-donor. Methylation of this position is thought to favour streptomycin binding (indicated as thick arrow).

Various non-synonymous single nucleotide polymorphism (nsSNPs) in the inhA gene and the gidB (glucose-inhibited division gene B) gene are associated with resistance to isoniazid and streptomycin respectively. The inhA gene encodes the protomers of the enoyl-[acyl-carrier-protein (ACP)]-reductase enzyme (InhA), which oligomerise to form a homotetramer. The enzyme catalyses an essential step in the type II fatty acid synthesis (FASII) pathway, required for biosynthesis of mycolic acid, a crucial component in bacterial cell walls [7]. Unlike other components of the FAS-II system, only one enoyl-ACP reductase is found in Mtb [8], [9]. As such, InhA is an essential enzyme. Isoniazid is a prodrug, which is converted to the active, radical form by the KatG enzyme [10]. The isonicotinic acyl radical can spontaneously react with NAD(P)H/NAD(P)+ to form a covalent adduct, INH-NAD(P) (Fig. 1). INH-NAD(P) adducts have been shown to form complexes with at least 18 proteins [11], [12], and as such, the full extent of interactions that contribute to isoniazid action are unclear. Nonetheless, the primary known target of isoniazid is InhA, which binds with high affinity to INH-NAD(P). INH-NAD(P) occupies both the NADH binding site and the substrate binding site, acting as a competitive inhibitor of both NADH and substrate binding to InhA (Fig. 1). Fig. 2.

Created using Biorender [13].

However, the mechanism by which isoniazid resistance can arise from mutations in InhA is unclear. Early studies showed that certain InhA mutations known to be associated with isoniazid resistance raised the disassociation constant (K_D_) of NADH binding to InhA. It was therefore proposed that mutations might also reduce binding of INH-NAD, such that it could no longer outcompete NADH for binding to InhA [7], [9], [14], [15]. However, in 2003, Rawat and colleagues found that binding of INH-NAD to InhA was not significantly affected by these mutations, despite the reduction in NADH binding [16]. In light of this, they proposed that mutations may inhibit INH-NAD binding only within the context of heterotypic protein-protein interactions of InhA, possibly with other components of the FASII pathway. This theory is supported by affinity chromatography and two-hybrid experiments that have shown InhA to interact with several other bacterial enzymes, including MabA and DHFR that are also inhibited by INH-NAD(P)H [11], [17], [18], [19], [20]. Thus, the current evidence points to allosteric modulation of InhA through protein-protein interactions being key to the mechanism by which mutations can cause drug resistance, although the nature of these protein-protein interactions is very much unclear.

The gidB gene encodes for the non-essential, ribosomal RNA small-subunit methyltransferase G (GidB), a monomeric enzyme that uses an s-adenosyl-methionine (SAM) substrate as the methyl-donor group [21]. The enzyme specifically methylates the N7 position of G518 of the 16 S rRNA, part of the ‘530 loop’ which forms the A-site pocket [22]. Crystal structures of streptomycin bound to the bacterial 30 S ribosomal subunit have shown that the drug binds to the phosphate backbone of the 16 S rRNA at positions within and nearby the A-site, including m^7^-G518 [22], [23], [24], [25], [26]. It has been found that in streptomycin resistant GidB mutants, position G518 of the 16 S rRNA is no longer methylated. This was thought to reduce binding of streptomycin to the 30 s ribosome [21], [22], [27], [28], [29], [30]. One study using *E. coli* homology modelling found that certain mutations appeared to alter the structure of the SAM binding site, explaining the loss of GidB function observed in streptomycin resistance-associated mutants. However, this has yet to be verified using an Mtb structure of GidB, which became available only in 2021.

In this study, we used *in silico* predictions and analyses to better understand the actions of 11 isoniazid and 79 streptomycin resistance-associated nsSNPs in InhA and GidB protein structures respectively (see Materials and Methods). Various bioinformatics tools including SDM [31], mCSM-lig [32], AlloSigMA [33], and AutoDock Vina [34] were employed to study the impact of resistance-associated mutations on protein stability, ligand affinity, allosteric modulation and ligand binding respectively. SDM is a knowledge-based approach, which uses information on how protein substitutions are tolerated in structural environments [35] to produce a predicted stability score for the wild-type and mutant structures (pseudo ΔG_folding_). The difference in pseudo ΔG_folding_ (ΔΔG_folding_) between wild-type and mutant structures indicates how a specific nsSNP affects protein stability. mCSM-lig uses a supervised machine learning algorithm, trained on thermodynamic datasets, to predict the difference in the log-fold change of ligand binding free energy between wild-type and mutant structures (ΔΔG_lig_). Both ΔΔG_folding_ and ΔΔG_lig_ are unitless quantities. AlloSigMA implements a structure-based statistical mechanical model of allostery (SBSMMA) framework [36], which accounts for global protein dynamics and changes in local elastic energy. For each residue, the server averages over all the possible neighbour conformations in the original and perturbed state, allowing for calculation of the change in the allosteric free energy (ΔG^mod^) of any residue in the structure as a result of a specific perturbation defined by a mutation. AutoDock Vina is molecular docking software which uses an iterated local search global optimizer in combination with knowledge-based potentials and empirical scoring functions to predict favourable poses for ligand binding to a protein [34].

## 2 Results

Overall, the results presented here show that InhA resistance-associated mutations disfavour NADH binding, without affecting INH-NAD binding to InhA. This suggests that resistance is not due to an inability of INH-NAD to bind InhA (in isolation). However, all mutations were predicted to allosterically modulate F158, F149 and W222, which form key interactions specifically with INH-NAD. Allosteric modulation of these residues may be key to the resistance mechanism. All GidB mutations were predicted to disfavour substrate binding, explaining the loss of G518 methylation in mutants. It is likely that these mutants disrupt specific protein-ligand binding interactions by altering the ligand binding site. The relationship between ΔΔG_lig_ and ΔΔG_folding_ was also investigated. Based on the data available, there was insufficient evidence to support a relationship existing between ΔΔG_lig_ and ΔΔG_folding_. However, it was noted that if three highly destabilising mutations were excluded a strong positive correlation was present for the set ΔΔG_folding_< −2.5. Below, we discuss the results in detail.

### 2.1 Isoniazid resistance-associated mutations

#### 2.1.1 Effects of drug-resistance associated mutations on InhA folding

**Fig. 3:**
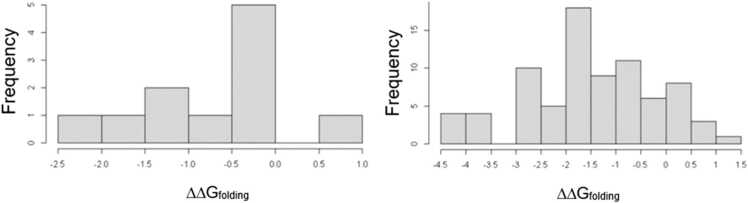
Histograms showing distribution of predicted ΔΔG_folding_ for InhA mutants (left) and GidB mutants (right).

The effects of resistance-associated mutations on protein stability were studied using SDM [31] (Supplementary Materials). The predicted ΔΔG_folding_ values for InhA mutations were distributed with a mean of − 0.651 and a range of 3.02 (S^2^ = 0.690) (Fig. 3). All of the InhA mutations were predicted to weakly destabilise protein folding (−2.5 ≤ ΔΔG_folding_ **<** 0.0), except *S94A*, which was predicted to be stabilising (0.0 *<* ΔΔG_folding_ ≤ 2.5). However, none was predicted as highly destabilising (ΔΔG_folding_ *<*-2.5) or highly stabilising (ΔΔG_folding_ *>* 2.5).

#### 2.1.2 Binding affinities of cofactor (NADH), inhibitor (INH-NAD) and substrate (THT)

The predicted effects of mutations on ligand binding to InhA are shown in Table 1. For binding of the inhibitor (INH-NAD) and the substrate trans-2-hexadecenoyl-(N-acetylcysteamine)-thioester (THT), the predicted ΔΔG_lig_ values for all mutants fell within the neutral range (−0.5 <ΔΔG_lig_<0.5). On the other hand, all mutations were predicted to considerably reduce binding affinity of NADH (ΔΔG_lig_<−0.5). The ΔΔG_lig_ values for NADH binding were distributed with a mean of − 1.29 and a range of 0.26 (S^2^ = 0.008), reflecting the small spread in the data. However, it was noted that the range of 0.26 corresponded to the difference in ΔΔG_lig_ between mutants of the same residue (*S94A* and *S94L*). These predictions suggest that none of the isoniazid-resistance associated mutations analysed in this study act to disfavour binding affinity of the INH-NAD adduct over NADH. No significant correlation was found between the ΔΔG_folding_ values and ΔΔG_lig_ values for NADH binding resulting from mutation.

**Table 1:** mCSM-lig predictions for ΔΔG_lig_ of INH-NAD, NADH and THT binding to InhA (units in log affinity fold change). The predicted ΔΔG_lig_ values for all mutants of INH-NAD and THT binding fell within the neutral range (−0.5 <ΔΔG_lig_<0.5).

	Predicted ΔΔG_lig_		
Mutation	INH-NAD	NADH	THT
S94A	-0.44	-1.48	-0.01
I95P	-0.51	-1.46	-0.12
V78A	-0.38	-1.31	0.03
I16T	-0.22	-1.28	0.29
I95T	-0.22	-1.28	0.29
I194T	-0.22	-1.28	0.29
I21V	-0.02	-1.25	0.03
K8N	-0.36	-1.25	-0.27
I21T	0.07	-1.22	-0.2
I47T	0.07	-1.22	0.44
S94L	0.02	-1.22	0.33

#### 2.1.3 Mutations allosterically modulate protein structure

**Fig. 4:**
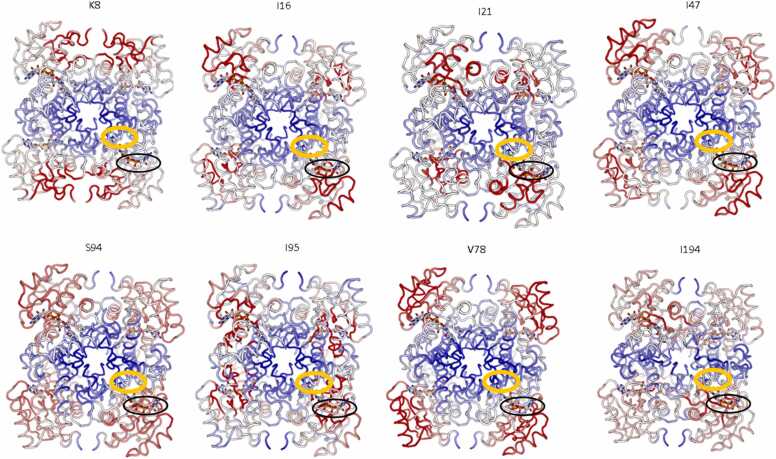
The effects of perturbation of residues K8, I16, I21, I47, V78, S94, I95 and I194 on conformational change within the structure of InhA:INH-NAD. Gradation from red-white-blue corresponds to lowest-neutral-highest ΔG^mod^ (decreased-neutral-increased conformational change) respectively (within a particular structure). Perturbation of all residues was predicted to promote conformational change at the protein-protein interface. Perturbation by all residues except I194 promoted conformational change in the substrate binding site (circled orange). Perturbation of all residues except K8 restricted conformational change in the cofactor binding site (circled black).

The allosteric sensitivity (ΔG^mod^) of each residue in the structure in response to perturbation of residues K8, I16, I21, I47, V78, S94, I95 and I194 was predicted for the InhA:INH-NAD complex and for an InhA:NAD:THT (substrate) complex. No significant difference was found between the predictions for these two structures (p*>*0.05). However, the results showed that perturbation of any one among this set of resides led to allosteric modulation of other residues in this set - mostly to restrict conformational change (ΔG^mod^*<*0.0 kcal/mol) (Table 2). Moreover, the results showed that mutation of all residues (except K8) were predicted to restrict conformational change in the NADH binding site of InhA (Table 3). Perturbations of all residues were found to promote conformational change at the protein-protein interface, and (with the exception of I194) the substrate binding site (Fig. 4). It was also noted that perturbation of all residues lowered the ΔG^mod^ of F41, while raising the allosteric free energy of F149, W222 and Y158 (ΔG^mod^ *<* 0.0 kcal/mol). This was with the exception of I194, which actually lowered the allosteric free energy of F149 and W222, but did raise that of Y158.

**Table 2:** Allosteric free energy change (kcal/mol) resulting in residues K8, I16, I21, I47, V78, S94, I95, I194 from perturbation of a different residue in this set. Cell shading indicates relative change in ΔG^mod^, with gradation from red-white-blue corresponding to lowest-neutral-highest ΔG^mod^ (decreased-neutral-increased conformational change).

**Table 3:** Allosteric free energy change (kcal/mol) predicted for substrate binding site and cofactor binding site (average of all residues) and for specific residues F41, F149, Y158 and W222. Cell shading indicates relative change in ΔG^mod^, with gradation from red-white-blue corresponding to lowest-neutral-highest ΔG^mod^ (decreased-neutral-increased conformational change)*.*

### 2.2 Streptomycin resistance-associated mutations

#### 2.2.1 Effects of drug-resistance associated mutations on GidB folding

The predicted stability values for the GidB mutations were distributed with a mean of − 1.48 and a range of 5.85 (S2 =1.680), reflecting that they were overall more destabilising than InhA mutants, and had a greater range of values (Fig. 3). Of the 79 GidB mutations studied, one was predicted to be neutral (ΔΔGfolding = 0.0) and 11 were predicted as weakly stabilising. The remaining 67 mutations were predicted to be either weakly or highly destabilising (ΔΔGfolding<0.0) and 17 of these were predicted to be highly destabilising, suggesting that these mutations considerably impact protein stability.

#### 2.2.2 Molecular docking and drug binding

**Fig. 5:**
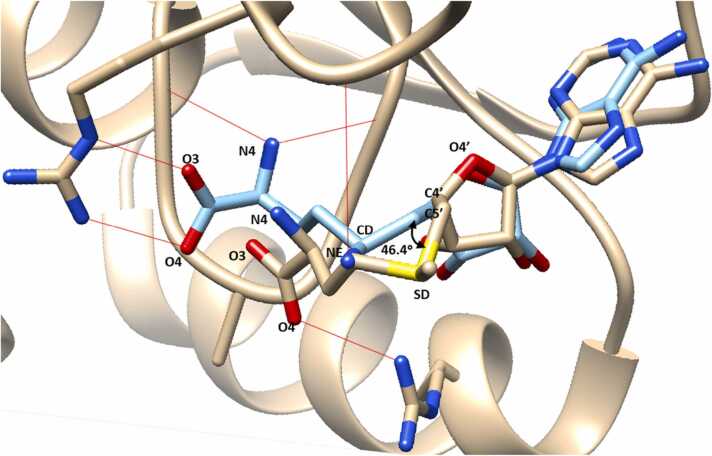
Comparison of predicted docking pose of SAM (gold) and known binding position of SFG (cyan). A 46.4° rotation along the C4′-C5′ bond causes structural variation between SAM and SFG. H-bonds in red were found not to be common between the two structures.

In order to predict how mutations might affect substrate binding to GidB, molecular docking of GidB:SAM was carried out, using AutoDock Vina [34]. Ten docking models were obtained. The highest affinity pose (−7.2 kcal/mol) was selected and binding of SAM was compared with the known binding position of substrate analogue sinefungin (SFG), all atom RMSD = 5.6 Å (Fig. 5). The O4′-C4′-C5′-SD torsion in SAM was found to be − 156.3° whereas the O4′-C4′-C5′-CD torsion angle in SFG was found to be 157.3°. Structural variation was found to affect H-bonding interactions of common atoms N4, O4 and O3.

#### 2.2.3 Binding affinity of SAM to GidB is reduced by all mutations

**Fig. 6:**
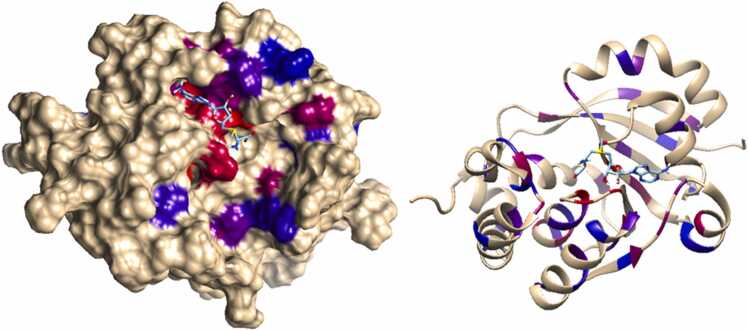
Surface (left) and cartoon (right) representations of the relative magnitude of predicted ΔΔG_lig_ values as a result of mutation at coloured residues (for residues where multiple mutations occur average value was taken). Gradation from red to blue represents lowest to highest ΔΔG_lig_ value. Mutations in the SAM binding site are shown to disfavour ligand binding the most.

All mutations reduced the binding affinity of SAM for GidB. The ΔΔG_lig_ predictions for SAM binding were distributed with a mean of − 1.14 and a range of 0.875 (S^2^ = 0.034), reflecting the relatively low spread in the data, with the exception of the most destabilising mutant, G71E, for which the predicted ΔΔG_lig_ was found to be 0.25 lower than the next most destabilising mutant. Mapping of the ΔΔG_lig_ prediction values for each residue onto the structure of the GidB protein (Fig. 6) showed that the mutations with the highest ΔΔG_lig_ for ligand binding were found near the ligand binding pocket.

**Fig. 7:**
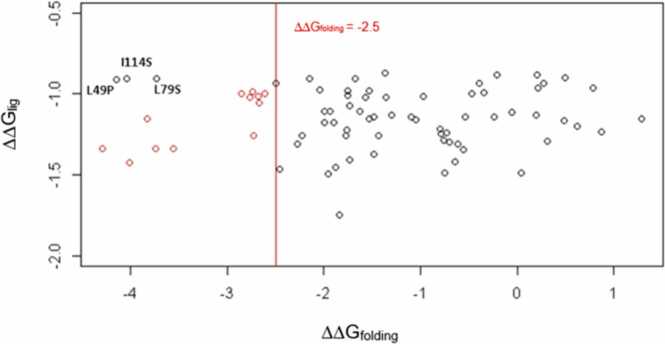
Plot of ΔΔG_folding_ against ΔΔG_lig_. Points in red represent highly destabilising mutants with the exception of L49P, I114S and L79S. Note that three results are superimposed as they have the same values (V88A, V65A, V202A).

To determine whether the predicted reduction in ligand binding was related to disfavoured protein stability, the SDM and mCSM-lig predicted ΔΔGs for each mutation were compared. No significant correlation was noted for the data as a whole (ρ = 0.01, p = 0.92). The data were then separated into sets of SDM ΔΔG_folding_ *<* −2.5 (highly destabilising) and SDM ΔΔG_folding_ *≥* −2.5, and the correlation in these two sets was calculated. A weak correlation was observed for the set ΔΔG_folding_ ≥ −2.5 (ρ = 0.13, p = 0.32) and the set ΔΔG_folding_< −2.5 (ρ = 0.12, p = 0.65) but both were found to be insignificant. It was noted that if three mutants (L49P, L79S and I114S) with the lowest ΔΔG_lig_ values from the set ΔΔG_folding_< −2.5 were excluded from the analysis, a moderate-strong correlation was present (ρ = 0.65, p = 0.01). However, since the mCSM-lig values for these mutants were found to be only 1.3 standard deviations from the mean (for set ΔΔG_folding_<−2.5) they were not classed as outliers (≥ 2 standard deviations from the mean). Fig. 7.

## 3 Discussion

### 3.1 Allosteric changes in the InhA structure may be involved in resistance

It is currently thought that isoniazid resistance may involve heterotypic protein-protein interactions in vivo [16]. It has been suggested that isoniazid resistance-associated mutations in InhA may act by disrupting these interactions in the INH-NAD bound protein [37]. However, our results show no significant difference between the predicted allosteric sensitivity of the InhA:INH-NAD and the INH:NAD:THT complex to isoniazid-resistance associated mutations. This is not consistent with the possibility that mutations disrupt protein-protein interactions with the inhibitor-bound structure, since if this were the case the allosteric response of the inhibitor-bound structure to these mutations would be expected to differ from the substrate-cofactor-bound structure.

However, we found that in these structures, perturbations of residues K8, I16, I21, I47, V78, S94, I95 and I194 were generally predicted to restrict conformational change within the cofactor binding site and promote conformational change in the substrate binding site and at the protein-protein interface (Fig. 4). The similarities in the allosteric response of the InhA structure to perturbation of these residues suggest that these mutations may act by the same mechanism. On the other hand, it was noted that perturbation of K8 increased allostery in the cofactor-binding site, and that mutations in I194 reduced allostery within the substrate binding site, although they still were found to modulate key residues F41 and Y158 as with the other mutants. Current literature does not cite the minimum inhibitory concentration (MIC) values for many of the mutants studied here. These datasets would facilitate future experiments to investigate whether the predicted differences in allosteric modulation of the structure by I194 and K8 correspond to differences in the MIC of isoniazid between these mutants and the others. Additionally, experiments to predict whether neutral mutations (also not currently characterised in the literature) allosterically modulate the structure in the same way would also confirm whether the trends observed here are specifically linked to resistance.

**Fig. 8:**
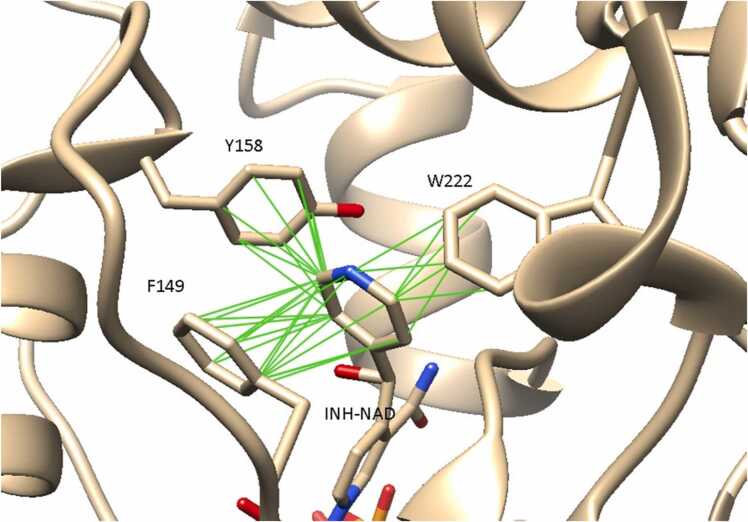
Hydrophobic contacts formed between residues F149, Y158 and W222 specifically with INH-NAD. Contacts (green) shown between atoms with VDW overlap ≥ - 0.5 Å.

Nevertheless, these findings suggest that allosteric modulation of the structure resulting from mutations could be important to the mechanism by which resistance is caused. Particularly of note is the finding that in all mutations (including I194), the allosteric modulation range of F41 was reduced whilst those of F149, W222 or Y158 were increased. These residues form key interactions with NADH and INH-NAD in the cofactor binding site and with INH-NAD in the substrate binding site (Fig. 8). Moreover, the observation that perturbation of any one of the residues mutated in isoniazid-resistant strains led to allosteric modulation of other such residues (Table 2) is further conducive to the idea that these mutations act by the same mechanism, and that allosteric modulation of the InhA structure resulting from these mutations plays a role in the mechanism of isoniazid resistance. Based on the findings, we speculate that in vivo mutations may act to prime these residues for additional conformational changes upon protein-protein interactions, upon which INH-NAD binding might be disrupted. Further experiments to elucidate the nature of these protein-protein interactions could help to shed more light on this.

**Fig. 9:**
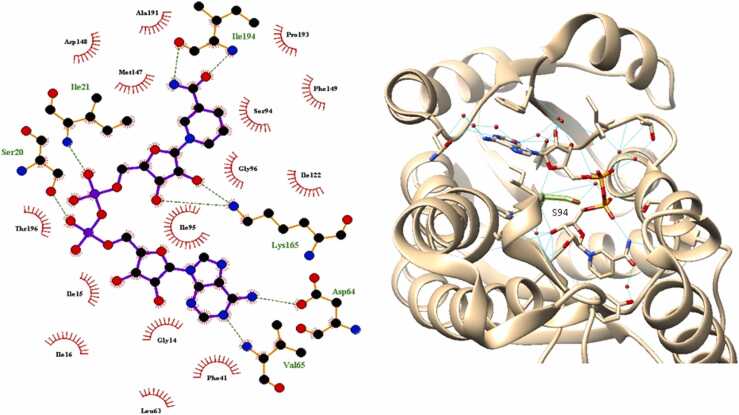
Left (a) direct hydrophobic and hydrogen bonding protein-ligand contacts formed between InhA and NADH. The contact diagram was generated using LIGPLOT [40]. Right (b) water-mediated hydrogen bonding network between binding-site residues and ligand. S94 forms part of this network.

However, there were limitations associated with modelling the allosteric effects of isoniazid resistance-associated mutations in InhA. Crystal structures of NADH and INH-NAD bound to the active site of InhA show that a network of water-mediated H-bonding interactions is formed between the ligand and active site residues (including S94) [9] (Fig. 9). However, the Allosteric Signaling and Mutation Analysis (AlloSigMA) server does not account for water molecules when making predictions, meaning that any role that these H-bonding interactions might play in allosterically modulating the structure could not be modelled. A further limitation was that the Structure-Based Statistical Mechanical Model of Allostery (SBSMMA) model [36] used by the AlloSigMA server is equipped only to predict the change in residue-residue contacts resulting from an UP mutation (substitution with bulky residue) or DOWN mutation (substitution with Ala/Gly). However, the majority of isoniazid resistance associated mutations cannot be appropriately modelled in this way, since many mutations (e.g. I → T) involve changes in polarity and insufficient changes in residue size to be considered UP or DOWN.

Due to these limitations, the modulation range (ΔG^mod(ij)^ = ΔG^UPij^-ΔG^DOWNij^), which “characterizes how significantly mutations on residue i affect the dynamics of a responding residue j” [36], was used to model the effects of perturbation of residues associated with isoniazid-resistant mutants. As such, the predictions in this study do not model the specific effects of mutation to a particular residue, but rather give an idea of how strongly perturbation of a residue is able to affect the allosteric free energy of the other residues. Despite these limitations, the predicted increase in conformational free energy of residues at the protein-protein interface is consistent with findings that mutants I21V, I47T and S94A induce conformational change at the dimer-dimer interface [37]. Moreover, crystal structures determined of InhA mutants *S94A* and *V78A* bound to INH-NAD found considerable changes in the sidechain torsion angles of residues Y158, F149, W222, and F41 in comparison to the WT [38]. These results are consistent with the findings presented here.

### 3.2 Isoniazid resistance associated mutations reduce binding affinity for NADH

Isoniazid resistance-associated mutations were found to disfavour binding of NADH to InhA. However, binding affinities of INH-NAD and the substrate THT were not affected by mutations. This is consistent with previous studies that have found mutations *S94A, I21V* and *I47T* to significantly inhibit binding (increased K_D_) of NADH to InhA but had little effect on the kinetic parameters of INH-NAD or substrate binding [15], [16]. Our findings suggest that this is also the case for mutations K8N, I194T, I21T, V78A, I95P, I95T, S94L, and I16T.

No correlation was found between the predicted ΔΔG_folding_ and the ΔΔG_lig_ for binding of NADH. This suggests that reduced ligand binding is not related to the predicted weakly destabilising effects of mutations. This is consistent with observations that the crystal structures of mutants I21V, I47T and S94A showed no significant differences in secondary structure or backbone conformation when compared with the wild type (WT) [39]. Instead, the predicted ΔΔG_lig_ values can be understood to be due to the disruption of specific residue-ligand contacts, and are consistent with observations from crystal structures of WT and mutant InhA:NADH. Residues I16, I21, I95 and I194 are all located in the NADH binding site of InhA (Fig. 9a), and all form hydrophobic contacts with the ligand. As such, I → T/V/P mutations can be understood to disrupt these protein-ligand interactions, and this has been shown to occur in the NADH-bound crystal structure of I21V [39].

Moreover, S94 forms part of the water-mediated network of H-bonds between the ligand and binding site (Fig. 9b). These H-bonding interactions have been shown to be disrupted in the S94A mutant structure [38], [39]. It was noted that whilst S94A was predicted to destabilise NADH binding the most, S94L was predicted to destabilise binding the least. The difference between the predicted ΔΔG_lig_ values for these two mutations may be due to the fact that in mutation S → L, the loss of H-bonding interactions is compensated for by increased hydrophobic contact with the ligand.

Mutations of residues I47, V78 and I194, located away from the binding site, were also predicted to reduce ligand-binding affinity through allosteric modulation of binding site, disrupting hydrophobic and H-bonding interactions. Isoniazid resistance-associated mutations restrict conformational change of residues I16 and I95 (Table 2), and all mutations restrict conformational change of F41 (Table 3). It has been shown that in the wild-type structure, F41, I16 and I95 undergo conformational changes to accommodate binding of NADH [41]. In particular, F41 forms π-stacking interactions with the adenine moiety of NADH. Thus disfavouring binding of NADH in isoniazid-resistance-associated mutants could also be explained by reduced conformational change to accommodate NADH binding.

### 3.3 Isoniazid resistance-associated mutations do not reduce binding affinity of the inhibitor

The binding positions of the cofactor and inhibitor in the NADH binding site are superimposable, except that INH-NAD occupies part of the substrate binding site with its aromatic C4N substituent [9]. It therefore follows that the difference in ligand-binding affinity in response to mutations is linked to this substituent. Studies comparing the crystal structures of InhA bound to NADH and INH-NAD found the only major structural difference to be the displacement of F149 to form pi-stacking interactions with the C4N aromatic substituent of INH-NAD [9]. Additionally, the crystal structure of InhA bound to INH-NAD also shows that 2 additional aromatic sidechains, W222 and Y158, are located within 4 Å of this aromatic substituent (Fig. 8). The aromatic sidechains of these residues could stabilise INH-NADH binding to InhA, by contributing hydrophobic (edge-face stacking) interactions with INH-NAD. Thus, while isoniazid resistance-associated mutations can be understood to result in the loss of interactions with both NADH and INH-NAD, it is likely that these contacts are not crucial for INH-NAD binding. Instead, INH-NAD binding may be stabilised by specific (stacking) interactions formed by the C4N substituent of INH-NAD with residues F149, Y158 and W222 in the substrate binding site.

### 3.4 All GidB mutants act to disfavour ligand binding

A model of the GidB:SAM complex obtained by molecular docking was used to predict the effects of streptomycin resistance-associated mutations on SAM binding to GidB. SAM was found to dock at the same site as the substrate analogue, SFG, but there was variation in the conformation of the two ligands and their interactions (Fig. 5). It appears that the structural variation is mainly due to the 46.4° difference in the O4′-C4′-C5′-SD torsion angle of SAM and the O4′-C4′-C5′-CD torsion angle in SFG. The additional H-bond formed to NE in SFG could explain this difference.

All streptomycin resistance-associated mutants are predicted to destabilise substrate binding to GidB. This suggests that the loss of methylation of ^7^N-G518 of the bacterial 16 S rRNA observed in these streptomycin resistant mTB strains is a result of decreased binding affinity of the methyl-donor substrate to the methyltransferase GidB enzyme. In a separate study [42], structural variation in GidB mutants (L108R, L160R, L16R, V89M, S149R, T146M, G76C, R187, V171I) was predicted based on *E. coli* homology modelling. This study qualitatively analysed structural changes in the binding sites of these mutants, concluding that these changes likely disrupt SAM binding.

The results presented here quantitatively confirm that SAM binding is disrupted in these mutants. Additionally, the results allow for the relative effects of mutants on ligand binding to be quantified. Further experiments to compare the ΔΔG_lig_ values against the MICs for the mutants studied here could confirm reduced substrate binding as the cause for resistance.

### 3.5 Highly destabilising GidB mutants are located within key structural motifs

**Fig. 10:**
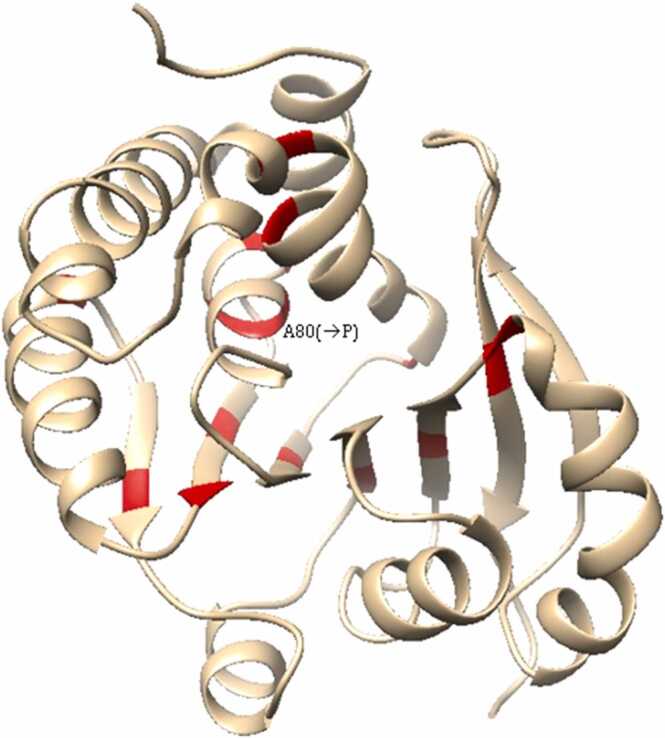
Location of highly destabilising mutants in GidB. Highly destabilising mutations occur within the hydrophobic core of the protein and may destabilise the secondary and tertiary structure, particularly the 7-stranded β-sheet (e.g. A80P).

Our analysis of 17 streptomycin resistance-associated mutations in GidB predicts their strong destabilisation of the protein (ΔΔG_folding_ *<* - 2.5). The crystal structure of GidB (PDB ID 7CFE) shows that, in the WT-protein, these residues are located within the core of the structure. They form hydrophobic interactions that stabilise secondary structural motifs, in particular the β-7mer, and their organisation into the tertiary structure *(*Fig. 10*)*. The mutant A80P (ΔΔG_folding_ = −4.29) is predicted to impact the protein folding the most, severely disrupting both the secondary and tertiary structure. This mutation restricts the peptide-backbone conformation, destabilising the formation of the α-helix, and causing steric clash at the interface between the α-helix and 7-mer β-sheet structure *(*Fig. 10*)*. Similar disruption to the structure can be understood to occur from mutations L49P, L91P, L59P, A133P and A82P (−4.15 <ΔΔG_folding_<−3.56).

Additionally, hydrophobic interactions formed between the secondary structural motifs would be lost by mutation of residues L160 (→R), W45 (→S), G164 (→C), A134 (→E) and L108 (→R) to polar/charged residues, and mutations of resides V88, V65, V202 (→A) and V65 (→G) to smaller residues. These mutants were generally less destabilising (- 2.85 <ΔΔG_folding_<−2.61) than mutations to proline. However, two hydrophobic to polar mutations, I114S (ΔΔG_folding_ = −4.04) and L79S (ΔΔG_folding_ = −3.73) were predicted to be considerably more destabilising than other such mutants.

### 3.6 Mechanisms for destabilisation of ligand binding in drug resistance

It was found that the mutants with the most negative ΔΔG_lig_ were located within or nearby to the binding site (Fig. 6). However, overall, low variation was observed in the ΔΔG_lig_ results (S^2^ = 0.034), suggesting that most mutants had relatively similar impacts on ligand binding. The aforementioned homology modelling study found that in proteins with mutations L108R, L160R G76D, S149R, V89M, T146M, G76C and L16R (also studied here), the structure of the ligand binding site was considerably altered [42]. This study also indicated that GidB is a relatively small protein, whose “active site consists of 16 interacting amino acids spread over the entire protein sequence,” meaning that the binding-site is highly vulnerable to mutations. Based on the findings presented here, it is therefore likely that all of mutants act to disrupt the binding site structure. Additional experiments to model these mutants *in silico*, or experiments to determine their crystal structures could confirm this.

We speculated that for some destabilising mutations reduced ligand binding affinity may be proportional to protein destabilisation since the reduction in ligand binding affinity is a direct result of destabilised protein folding (i.e. mutations act at a global level – mechanism A). In other cases the mutation may act to disrupt specific protein-ligand interactions at the ligand binding site, without considerably affecting protein stability (mechanism B). It may also be possible that a mutant acts to disfavour ligand binding and affects protein stability, but that these two effects are not linked (mechanism C). Highly destabilising mutants are more likely to cause major structural changes (which could directly contribute to reduced ligand binding) than weakly destabilising mutants. Thus, a larger proportion of these might be expected to act via mechanism A than weakly destabilising mutants.

To explore this hypothesis we tested for correlation between ΔΔG_folding_ and ΔΔG_lig_ in the sets ΔΔG_folding_< −2.5 (highly destabilising) and DDG_folding_ ≥ 2.5. While no significant correlation was observed for either sets, it was noted that if three mutants were excluded from the set of highly destabilising mutants, a significant correlation was observed (ρ = 0.65, p = 0.01). It is conceivable that mutants I114S, I17S and L49P act via mechanism C, whilst the remaining highly destabilising mutants act globally (mechanism A). However, since the criterion for outliers was not met, there was no statistical basis to exclude these points. Thus, the available data presented here provides insufficient evidence to support a relationship between ΔΔG_lig_ and ΔΔG_folding_ in the context of the sets analysed.

Nonetheless, further study into a possible relationship between ΔΔG_folding_ and ΔΔG_lig_ in the context of different sets, based on additional data, could provide more insight into this subject. Indeed, in the aforementioned modelling study [42] it was observed that for mutants L108R and L160R (ΔΔG_folding_ *<* −2.5), the overall structure of the binding cavity in the predicted structures appeared considerably more disrupted than in the other mutants modelled that are also studied here (ΔΔG_folding_ ≥ −2.5). This is consistent with the ideas presented above. Future *in silico* modelling studies to charaterise the structures of a greater number of the mutants studied here could help provide the additional data required to comprehensively investigate this topic.

## 4 Conclusion

Using *in silico* techniques, we have shown that, for the mutants studied, isoniazid resistance cannot be explained by disruption of protein folding or a reduction in INH-NAD binding to the protein in isolation, and we suggest that this may be due to stabilising interactions formed in the substrate binding site. Based on the finding that certain key residues in the cofactor and substrate binding sites may be allosterically modulated by isoniazid resistance-associated mutations, we argue that allosteric modulation of these residues may be important in resistance. However, the question of exactly how this allosteric modulation of the structure might lead to resistance remains to be conclusively answered. Additionally, we found that streptomycin resistance-associated GidB mutants can be understood to have reduced binding affinity for the substrate SAM, and suggest mechanisms by which they may act.

It is important to note that the *in silico* techniques used in this study are limited in their prediction capabilities. This has already been discussed for AlloSigMA, but SDM and mCSM-lig are also limited by the datasets that they use in making their predictions. As such, further, independent *in silico* and in vivo experiments, mentioned throughout the discussion, would be crucial for verifying the results presented and further exploring the conclusions made. Nonetheless, these findings contribute to a more detailed understanding of the mechanisms by which resistance to these drugs occur and could be valuable for directing the development of future treatments, as well as improving those currently available.

## 5 Materials and methods

### 5.1 Structure retrieval

Structures were retrieved from the Protein Data Bank [43] for experimental analysis (Table 4).

**Table 4:** Structures retrieved and their experimental uses.

Structure	PDB ID	Analysis
InhA (Apo)	4TRM	SDM input
InhA:NAD:THT	1BVR	AlloSigMA input mmCSM-lig input
InhA:NADH	2AQ8	mmCSM-lig input
InhA:INH-NAD	2IDZ	mmCSM-lig input AlloSigMA input
SAM	SAM	Molecular docking
GidB:SFG	7CFE	SDM input Molecular docking

### 5.2 Mutation data

Raw data on drug resistance associated gene (DRAG) mutations in mTB inhA and gidB genes was obtained from DRAGdb (http://bicresources.jcbose.ac.in/ssaha4/drag/index.php) [44]. The data were refined to include only nsSNPs and checked against the experimental structures. The final dataset included 11 mutations in inhA and 79 mutations in gidB (Table 5).

**Table 5:** List of drug resistance mutations studied. Data was retrieved from DRAGdb.

Gene	Mutations
inhA	I16T, I21T, I21V, I47T, I95P, I95T, I194T, K8N, S94A, S94L, V78A
gidB	R187M, G34E, R96P, R47G, P75S, G28E, G164D, R83P, L160R, A134E, A80P, A138P, A183T, L49P, P93L, E92D, V202A, P93Q, V77G, V171I, L108R, V124A, L59V, A119D, A200E, L79S, A133P, E170D, R102P, G76R, W45S, T146M, V77A, G30R, G164J, R154W, P75R, L160H, V36G, L101F, L79W, N51D, V115G, V89M, S149R, S136P, R96J, L16R, G164R, P84J, A82P, G34A, V88A, E92K, G69D, F12L, H48Y, G76J, P75T, G71E, P84L, G117E, R206L, L128S, T146K, H48N, V65A, L94S, G76D, H48D, G71R, L94P, I114S, L59P, V65G, Q127P, L91P, L79F, A138V

### 5.3 Predicting the impact of mutation on protein stability

A standalone version of the SDM program [31], [35] was used to predict the effects of mutations on protein structure stability. SDM server (http://marid.bioc.cam.ac.uk/sdm2) is also available online for public use. It is worth noting that SDM uses environment specific substitution probability tables to express mutant stability score as unitless probability ratios described elsewhere [35]. Studies linking missense mutations with known phenotypes estimated that a stability margin of 1–3 kcal/mol can be accommodated without any immediate effect on the fitness of the protein [45], [46], [47]. Mutations causing severe disease phenotypes in Ig-like proteins are shown to destabilise proteins by> 2 kcal/mol [48]. From the structural point of view, disease causing mutations that affect protein function are more likely to be found buried in the protein core compared to the mutations on the protein surfaces [49], [50], [51], [52]. Previously, using the largest experimental mutant thermodynamic dataset we structurally classified highly destabilising (experimental ΔΔG_folding_<−2.5 kcal/mol) and highly stabilising mutations (experimental ΔΔG_folding_>2.5 kcal/mol) and showed that the highly destablising mutations were found in the protein interior characterised by the high residue packing density [31]. Based on the above studies and analysis, we categorized the SDM predicted mutant stability score (ΔΔG_folding_) into four types: weakly destabilising (−2.5 ≤ ΔΔG_folding_<0), highly destabilising (ΔΔG_folding_<−2.5), weakly stabilising (0 <ΔΔG_folding_ ≤ 2.5) and highly stablising (ΔΔG_folding_>2.5).

### 5.4 Predicting the impact of mutation on ligand binding

The mCSM-lig server (http://biosig.unimelb.edu.au/mmcsm_lig) was used to investigate the effects of drug resistance mutations on ligand binding affinity [32]. PDB files of the protein structures (Table 1) and text files containing appropriately formatted mutations lists were provided as input. It outputs a unitless score with positive and negative values of ΔΔG_lig_ corresponding to increased and reduced ligand binding affinities respectively.

### 5.5 Predicting the effects of mutations on allostery in InhA

AlloSigMA (http://allosigma.bii.a-star.edu.sg/home/) [33] was used to investigate the effects of drug resistance mutations on allostery. The allosteric free energy values (ΔG^mod^) can be used to represent either flexible (ΔG^mod^ *>* 0.0 kcal/mol) or restricted (ΔG^mod^ *<* 0.0 kcal/mol) conformational changes. PDB files of InhA bound to cofactor and substrate and to INH-NAD (Table 1) were provided as input to the server, and all four chains of the tetrameric assemblies were selected for analysis of allosteric signalling.

### 5.6 Molecular docking

Molecular graphics and analyses were performed with UCSF Chimera [53]. The SFG ligand was removed from the GidB structure. The resulting apo-structure was prepared for minimisation and docking. Molecular docking of SAM and gidB was carried out with AutoDock Vina. The docking search space was set to cover the whole protein. The ligand was treated as a flexible molecule, while the receptor was kept rigid. Lower AutoDock scores correspond to higher binding affinity. The highest affinity dock (−7.2 kcal/mol) was superimposed and aligned with the GidB:SFG structure to compare the binding positions of SAM and SFG.

## Declaration of Competing Interest

The authors declare the following financial interests/personal relationships which may be considered as potential competing interests: Arun Prasad Pandurangan is an Editorial Board member of the Computational and Structural Biotechnology Journal.
